# 

**Published:** 1885-07

**Authors:** 


### BOOKS AND PAMPHLETS RECEIVED.

Eleventh Annual Announcement of the American Veterinary College, 1885.

Annual Announcement of the New York College of Veterinary Surgeons and School of Comparative Medicine, Session, 1885-6.

Disinfection and Disinfectants. Preliminary Report made by the Committee on Disinfectants, of the American Public Health Association.

Annual Catalogue of the Michigan Agricultural College, 1884-5, Lansing, Mich.

Variations in the Form of the Beak, that takes place during its growth in the short-tailed Albatross. By Dr. R. W. Shufeldt, U.S.A., Fort Wingate, New Mexico.

Johns Hopkins University Circulars.

Annals et Bulletin de la Société de Medicine de Gand.

Journals—The Veterinarian, London; The Veterinary Journal, London; The Quarterly Journal of Veterinary Science in India; American Veterinary Review, New York; Der Zoologische Garten, Frankfort; Schweizer Archiv fur Thierheilkunde, Zurich; Archiv fur Wissenschaftliche und Praktische Thierheilkunde, Berlin; Wochenschrift fur Thierheilkunde und Viehzucht, Augsburg; Giornale di Anat Fisiol e Patol, degli Animali, Pisa; Il Medico Veterinario, Torino; La Clinica, Veterinaria, Milano; La Cronica Medica, Valencia; El Ensayo Medico, Caracas; Le Progres Medical, Paris; Kansas City Review of Science and Industry; The College and Clinical Record, Philadelphia; New England Medical Monthly, Sandy Hook; Nashville Journal of Medicine and Surgery; The Southern Clinic, Richmond; Chicago Medical Journal and Examiner; The Western Medical Reporter, Chicago; Journal of Cutaneous and Venereal Diseases, New York; Denver Medical Times; The St. Joseph Medical Herald; The Weekly Medical Review, St. Louis; The Polyclinic, Philadelphia; Medical World, Philadelphia; Texas Courier-Record, Fort Worth; The American Journal of Ophthalmology, St. Louis; Pacific Medical and Surgical Journal, San Francisco; New York Medical Journal; American Psychological Journal, Philadelphia; Leonard's Illustrated Medical Journal, Detroit; Kansas City Medical Index; The Therapeutic Gazette, Detroit; North-western Lancet, St. Paul; Indiana Medical Journal, Indianapolis; Horseshoer and Hardware Journal, Chicago; The Blacksmith and Wheelwright, New York; National Live Stock Journal, Chicago; The Breeder's Gazette, Chicago; Cultivator and Country Gentleman, Albany; Weekly Drover's Journal, Chicago; The Chicago Tribune; Jersey Bulletin, Indianapolis; Turf, Field and Farm, New York; The Canadian Breeder, Toronto.

